# Becoming a congenital heart surgeon: the long and challenging road

**DOI:** 10.1093/icvts/ivac250

**Published:** 2022-10-19

**Authors:** K A Jacob, N Hussein, A van Wijk, P P Heinisch, C Salih, L Galetti, J Hörer

**Affiliations:** University Medical Centre, Utrecht, Netherlands; Castle Hill Hospital, Cottingham, England, UK; University Medical Centre, Utrecht, Netherlands; German Heart Center Munich, Technical University of Munich, School of Medicine, Munich, Germany; University Hospital of Munich, Ludwig-Maximilians-Universität, Munich, Germany; Evelina Children's Hospital, Guy's & St Thomas' Hospitals, London, UK; Bambino Gesù Children's Hospital, IRCCS, Rome, Italy; German Heart Center Munich, Technical University of Munich, School of Medicine, Munich, Germany; University Hospital of Munich, Ludwig-Maximilians-Universität, Munich, Germany

**Keywords:** Congenital heart surgery, Training, Mentorship, Fellowship

## Abstract

Training in congenital cardiac surgery is potentially lengthier and more demanding than training in any other surgical field. The duration of training is proportional to the complexity of the specialization. The expertise of a wide range of procedures is required. There is no doubt that some individuals may acquire the requisite abilities with greater ease than others, but fundamentally, these are capabilities that can be taught and learnt. Moreover, congenital cardiac surgeons are required to have a detailed understanding of pathophysiology and morphology, in addition to the stamina and empathy required to manage these complex patients. A fellowship is just the start of such training and is followed by a long road eventually leading to a lifelong journey to become a qualified congenital cardiac surgeon. Effective mentorship is a prerequisite throughout training to guide surgeons on this journey.

## INTRODUCTION

In 1938, the first congenital heart procedure was performed when Richard Gross ligated a persistent ductal arteriosus. The world’s first open-heart operation using a heart–lung machine was performed to repair a congenital heart defect. John Gibbon closed an atrial septal defect (ASD) in Philadelphia in 1953. The management of congenital heart diseases (CHDs) has evolved considerably since. Advancements in the speciality have led to the majority of CHDs being amenable to either complete repair or palliation. Subsequently, the portion of children with these anomalies surviving to adulthood has increased with two-thirds of the CHD population composed of adults with congenital heart disease [1].

Congenital cardiac surgeons are a crucial pillar of cardiovascular care for both children and adults with the training pathway being lengthy and demanding. Despite the growth of congenital cardiac surgery worldwide, it is still not a recognized speciality in the majority of European countries. Consequently, associations like the World Society of Paediatric and Congenital Heart Surgery are striving towards clarifying training pathways and standardizing fellowships to assist the surgeon mobility between developed and developing countries [2].

## TRAINING

To become a congenital cardiac surgeon, one must often navigate a convoluted and heterogeneous training pathway with no guarantee of a consultant position on completion. In the majority of countries, medical education is followed by several years of residency. This usually consists of a 5- to 6-year training programme in adult cardiovascular or cardiothoracic surgery, which gives a solid foundation of the knowledge and surgical skill required for acquired heart surgery. Subsequently, senior surgical residents will then enrol into a congenital training curriculum for an additional 1–3 years [3]. The duration of each of these components may vary. For example, in Italy residents undergo 2–3 years of basic adult heart surgery training followed by a full 5-year congenital cardiac surgery programme. These programmes are generally based in academic, tertiary high-volume institutions.

Over the last 2 decades, many Western countries have introduced and/or standardized congenital cardiac surgery programmes to a more formalized and regulated specialty pathway [3, 4]. Prior to this training was heterogeneous and based on ‘learning by osmosis’ after years of shadowing senior surgeons in large volume centres. Although effective, this training was not protocolled nor provided consistent documentation of the basic elements of understanding and performance necessary for clinical practice. This practice still continues in Latin America, Africa and Asia [2]. In modern congenital programmes, surgeons must pass a standardized examination and are required to perform a set number of surgeries as the primary operator, to be accredited by their national medical council at the conclusion of their fellowship. These structured fellowship programmes have improved education and experience in congenital heart surgery, particularly neonatal surgery [4]. Nevertheless, heterogeneity remains and there is no international consensus over what a fellowship should entail. This lack of standardization hinders the global mobility of congenital cardiac surgeons and international collaboration, which adversely effects the provision of paediatric surgical care in numerous impoverished nations.

A disadvantage of most programmes is the lack of dedicated rotations in allied specialties to congenital cardiac surgery (i.e. interventional cardiology, echocardiography and critical care). To achieve clinical competence, congenital surgeons are required to have comprehensive factual knowledge and critical thinking skills in pre-, intra- and postoperative paediatric settings and longitudinal patient follow-up. The adult cardiac surgery postgraduate years alone are insufficient for neonatal or paediatric CHD diagnosis and care. Adding such rotations within these allied specialities would strengthen a congenital cardiac surgeon's clinical understanding and foster better collaboration between surgical, interventional, and medical specialties, which will potentially improve overall care quality. To tackle such issues, the European Association for Cardiothoracic Surgery (EACTS) Academy started an annual Fundamentals Course in Congenital Heart Surgery, which is aimed to address such knowledge limitations and provide trainee surgeons with an interest in CHD, a basic overview of essential knowledge required.

On the completion of fellowship training in congenital cardiac surgery, having had several years of postgraduate training, usually surgeons are merely able to perform simple procedures such as repair of septal defects, vascular rings and maybe a tetralogy of Fallot. Maturation to a fully qualified congenital cardiac surgeon proceeds gradually over several years of clinical practice under senior mentorship. Therefore, it is essential for young doctors to begin training in congenital cardiac surgery at an early stage, as it takes several years after certification to hone and acquire the abilities of a competent consultant. In contrast, the majority of adult-acquired cardiac surgeons are able to undertake straightforward coronary or valve surgery upon training completion. This factor combined with the prolonged training period in CHD possibly leads to the loss of potential excellent congenital cardiac surgeons. In the most recent poll conducted by the American Board of Thoracic Surgery, only half of participants in the American congenital heart surgery programme successfully completed their training and assessment [5]. These are issues that urgently need addressing.

## MORE THAN TECHNICAL SKILLS

Congenital cardiac surgery is a challenging and demanding specialty, which, as Dr Fraser has once put together, requires surgeons to have ‘elements of technical skill, judgement, diagnostic acumen, physical stamina and emotional intelligence’, to be successful [6]. In addition, patient and familial expectations are very high nowadays and excellent quality of care has become the standard. Newly appointed consultants are required to perform at the highest level from the start, unlike in the era of their mentors. Modern congenital heart surgery requires precision in an ever-more-difficult patient population, ranging from premature newborns with complicated multisystem abnormalities to adults with many comorbidities requiring multiple redo surgeries. In some countries, surgeon outcomes are accessible to the public adding further pressure to congenital cardiac surgeons.

Therefore, what would increase fellow satisfaction and thus assist training completion in this ever-increasing pressurized environment? Surveys from the USA and Europe have shown that more hands-on operative exposure and especially adequate mentorship lead to higher satisfaction in residents and fellows [7, 8]. Good mentoring facilitates the transition from fellow to consultant and the retention of surgeons. In the early consultancy/attending years, it is crucial to continue working in an environment surrounded by skilled surgeons and an enthusiastic, competent team of cardiac paediatric anaesthesiologists, cardiologists and intensivists to hone all of their necessary skills. The success of congenital cardiac surgeons is reliant on competent multidisciplinary teams as surgical outcomes are influenced by a variety of factors in addition to surgical performance. Working as a fellow surgeon with such a competent entourage would not only further build up the young surgeon’s technical skills but also ensure the maintenance of high competence and enhance their cognitive decision-making and emotional proficiencies. It would considerably make them more resilient and better prepared for the world of the above expectations and public gallery of complication rates.

## IDEAL TRAINING PROGRAMME

As described earlier, it is arduous to equip the next generation of congenital cardiac surgeons with the aforementioned competencies. Trainee surgeons are required to work under rules in duty hour schedules, which adversely effects the exposure trainee surgeons receive, compared to previous eras. On the other hand, benefits of such changes in working hours include the increase of females pursuing a career in cardiac surgery. Furthermore, mentor surgeons are confronted with the expectancy of excellent outcomes and publication of outcomes, which may further limit training opportunities. The recent scrutinization of patient morbidity outcomes, rather than only mortality, leaves minimal scope for surgeons to experience the learning curve. Therefore, a dilemma exists whereby training programmes are required to coordinate effective curricula in an era where the learning curve is no longer accepted.

Such modern constraints on surgical training have prompted training centres to adopt unique training approaches. These novel techniques include animal or three-dimensional-printed models, which offer trainee surgeons a risk-free environment in which to hone their technical surgical abilities prior to performing actual operations. This allows training centres to provide surgeons with better fellowships, while safeguarding their own institution from poor outcomes resulting from the steep learning curve. Despite these modern technologies, there are many who disagree and advocate rather the traditional approach of teaching residents by exposure and gradual hands-on experience. While each has advantages and weaknesses, we feel that they can be used synergistically to equip trainees with the aforementioned competencies. Three-dimensional or animals are excellent to train technical skills and procedural steps. Yet, these models cannot replace hands-on surgery since many of the subtle differences between adult and congenital cardiac surgery is due to the nature of the tissues [9]. Following training on such models, surgeons can hone these skills in the operating room. A good example of teaching by doing is demonstrated in a recent paper by Cleveland *et al*. They demonstrated a well-structured and successfully implemented training programme in California. It is based on 4 tenets: (i) mitigating the risk of surgery by careful case selection, (ii) allocation of cases in a fashion of graduated responsibility, (iii) avoiding the assignment of complex high-risk cases during training, and (iv) full commitment from all faculty members to the educational mission [10]. This programme led to no difference in mortality, morbidity or length of stay when similar cases were operated on by consultants or appropriately supervised residents. The caseload for residents included complex single-procedure neonatal surgeries, e.g. Norwood, arch repairs and truncus arteriosus procedures. These more complex procedures were performed in the second half of the programme with case selection chosen carefully (i.e. neonatal cases with few risk factors) [10]. All procedures were supervised by 2 scrubbed faculty members to reduce the operative risk and facilitate residents to perform these complex procedures. This graduated training programme with appropriate supervision is welcome and should be replicated by other programmes.

Mentorship by an experienced colleague is another vital aspect of an ideal training programme. Such a mentor is not merely a talented surgeon but also a committed coach and advisor for their mentees in all aspects of the congenital speciality. A successful mentor is one who creates a reciprocal and respectful relationship in a pleasant teaching environment both in and outside the operating room [11]. The ability to coach a resident through an operation in a calm and controlled manner is another important characteristic. Finally, attention to detail to ensure the best possible outcome for patients is admired by many [11, 12]. Unfortunately, structured and devoted mentor/mentee relationships are in short supply in the field of congenital heart surgery. This highlights the need to develop international internships to gain knowledge from various mentors. Advantages of training overseas include the opportunity to train in a different healthcare system with its unique facilities and practises, as well as the culture and people of the host country. On completion, surgeons can bring new ideas back to their own service after gaining knowledge of surrogate methods. An alternative solution would be to invite exemplary mentors to local training programmes to instruct residents on a variety of topics, not limited to surgical skill development.

Other competences such as clinical judgement and social skills in this demanding profession can be acquired by introducing rotations in specialities, which are vital for congenital cardiac surgery as a whole, such as cardiology and intensive care.

Last but not least, a successful training programme in congenital cardiac surgery can only be provided in high-volume centres. As the international nomenclature lists >150 different procedures, even the largest centres may rarely treat identical heart defects >25 times per year. The majority of procedures are performed <10 times per year. Therefore, residents should be exposed to the full breadth of the speciality on a regular basis within their training, which is only possible in larger centres.

## INTERNATIONAL PERSPECTIVE

Globalization has led to the proliferation of humanitarian programmes that provide congenital surgical care and training in the developing world [13]. The introduction of new therapies such as ventricular assist devices into the paediatric population along with the growth of heart transplantations due to the donor after circulatory deaths programmes in some territories will inevitably lead to an increase in procedures worldwide. This is in addition to the surging population of adults with CHD, who may require further redo surgeries in their lifetime. Data indicate that outcomes are improved when congenitally trained cardiac surgeons undertake these adult congenital procedures [14]. Consequently, the workload for young congenital cardiac surgeons has been increasing over the past few decades, with a recent survey revealing that half of current consultants work >70 h per week [7]. Nevertheless, a significant percentage of congenital cardiac surgeons would prefer to operate only on children and not on adults. A contentious consequence of this could be the emergence of the adult congenital cardiac surgeon. This new subspecialization of adult or congenital cardiac surgery would focus solely on complex redo operations and surgery on previously repaired congenital defects, i.e. coronary surgery on adult surgeons who had previous arterial switch surgery.

Furthermore, a large group of currently practicing surgeons are expected to retire within the next decade [7, 15]. A manpower analysis by the American Association of Thoracic Surgery has shown that the supply of cardiothoracic surgeons is falling consistently until 2030, concluding that the supply of congenital cardiac surgeons is being seriously affected [15]. Ergo, the need to train more congenital cardiac surgeons in the near future is crucial. Yet, continually teaching a large number of individuals with limited practical expertise is not an effective nor efficient solution to this challenge. It would be preferable to connect the number of trainees with the number of employment openings, including increasing the minimum case volume criteria. The emerging trend of regionalizing congenital heart surgery to high-volume centres is essential for such a fellowship design. As noted in the previous section, ideally only large institutions with a significant case volume would offer training programmes to train fellows by qualified mentors. In combination with this, it is advantageous to have a rotating timetable for authorized programmes to ensure a steady flow of newly educated surgeons. This could be done both nationally and internationally by organizations such as the EACTS, American Association of Thoracic Surgery or World Society of Paediatric and Congenital Heart Surgery to ensure there are enough qualified surgeons across each continent. For example, the European Board exams of the EACTS offer fellowship exams for those in congenital cardiac surgery training. This is aimed to standardize the knowledge of CHD across Europe and facilitate the mobility of surgeons across the continent [8]. However, this does not develop crucial skills required to be a competent congenital heart surgeon such as clinical decision-making and emotional intellect. To achieve this, fellows would be required to spend periods of their training abroad in high-volume and experienced centres across the continent or the Atlantic. Surgeons from developing countries could likewise benefit from such programmes, which could potentially lead to top-level congenital cardiac surgery becoming more available in their own countries.

## FUTURE

Despite the long and difficult road ahead, how do we attract the best and brightest students and residents to a career in congenital cardiac surgery? First and foremost, a trustworthy and accurate depiction of the difficult path to becoming a congenital surgeon must be provided. All countries should recognize the speciality of congenital cardiac surgery in order to provide proper and standardized training. Training programmes within congenital cardiac surgery should be developed to incorporate a high exposure of hands-on training within specialist centres and adopt novel methods of hands-on training. Mentorship has been identified as the most common motivator in surveys for pursuing such a vocation and should be fostered. However, due to the significant subspecialization of our profession, gaining access to congenital surgeons before interns develop their career interests remains difficult.

There are many more requirements than purely technical to become a successful congenital cardiac surgeon. This includes breadth of intellect, open-mindedness, adaptability, resilience, empathy and social skills, which are all crucial. Standardizing training programmes across countries, which incorporate key non-surgical rotations and encourage overseas training, will add to these fundamental skills. Mitigating risks with careful case selection and a committed staff of mentors for trainees is a prerequisite. Along these lines, international recognition of national training certification is a must to assist surgeon mobility and encourage collaboration. Now is the moment to implement these changes to develop excellent congenital cardiac surgeons, in the ever-changing world of medicine where publicly published quality has become the standard. We have a moral obligation to nurture the next generation of surgeons and make every effort possible to allow them to be successful while never compromising on the quality of care and patient well-being.

**Conflict of interest:** The authors report no potential Conflict of Interest for this article.

## Data Availability

No new data were generated or analysed in support of this research.

## References

[1] Marelli AJ , Ionescu-Ittu R, Mackie AS, Guo L, Dendukuri N, Kaouache M. Lifetime prevalence of congenital heart disease in the general population from 2000 to 2010. Circulation 2014;130:749–56. 24944314 10.1161/CIRCULATIONAHA.113.008396

[2] Tchervenkov CI , Herbst C, Jacobs JP, Al-Halees Z, Edwin F, Dearani JA et al Current status of training and certification for congenital heart surgery around the world: proceedings of the meetings of the global council on education for congenital heart surgery of the world society for pediatric and congenital heart surgery. World J Pediatr Congenit Heart Surg 2021;12:394–405. 33942697 10.1177/21501351211003520

[3] Mery CM , Kane LC. The ACGME fellowship in congenital cardiac surgery: the graduates' perspective. Semin Thorac Cardiovasc Surg Pediatr Card Surg Annu 2017;20:70–6. 28007069 10.1053/j.pcsu.2016.09.005

[4] Kozik DJ , Kogon BE. Impact of accredited training programs in congenital heart surgery. Semin Thorac Cardiovasc Surg 2020;32:492–7. 32433986 10.1053/j.semtcvs.2020.04.003

[5] Kogon B , Karamlou T, Baumgartner W, Merrill W, Backer C. Congenital cardiac surgery fellowship training: a status update. J Thorac Cardiovasc Surg 2016;151:1488–95. 27002229 10.1016/j.jtcvs.2016.02.039

[6] Fraser CD. Becoming a congenital heart surgeon in the current era: realistic expectations. J Thorac Cardiovasc Surg 2016;151:1496–7. 27016794 10.1016/j.jtcvs.2016.02.054

[7] Morales DL , Khan MS, Turek JW, Biniwale R, Tchervenkov CI, Rush M et al Report of the 2015 Society of Thoracic Surgeons congenital heart surgery practice survey. Ann Thorac Surg 2017;103:622–8. 27553498 10.1016/j.athoracsur.2016.05.108

[8] Cerqueira RJ , Heuts S, Gollmann-Tepeköylü C, Syrjälä SO, Keijzers M, Zientara A et al Challenges and satisfaction in Cardiothoracic Surgery Residency Programmes: insights from a Europe-wide survey. Interact CardioVasc Thorac Surg 2021;32:167–73. 33236099 10.1093/icvts/ivaa248 PMC8906668

[9] Mavroudis CD , Mavroudis C, Jacobs JP, DeCampli W, Tweddell J. Simulation and deliberate practice in a porcine model for congenital heart surgery training. Ann Thorac Surg 2018;105:637–43. 29275827 10.1016/j.athoracsur.2017.10.011

[10] Cleveland JD , Bowdish ME, Mack WJ, Kim RW, Kumar SR, Kallin K et al Resident education in congenital heart surgery does not compromise outcomes. J Thorac Cardiovasc Surg 2022;163:251–60. 33581904 10.1016/j.jtcvs.2020.12.112

[11] Sambunjak D , Straus SE, Marusić A. Mentoring in academic medicine: a systematic review. JAMA 2006;296:1103–15. 16954490 10.1001/jama.296.9.1103

[12] Cohen MS , Jacobs JP, Quintessenza JA, Chai PJ, Lindberg HL, Dickey J et al Mentorship, learning curves, and balance. Cardiol Young 2007;17:164–74. 18039410 10.1017/S1047951107001266

[13] Jonas RA. Presidential address: accomplishments and challenges ahead for congenital heart surgery. World J Pediatr Congenit Heart Surg 2011;2:202–10. 23804973 10.1177/2150135111398921

[14] Kogon BE , Plattner C, Leong T, Kirshbom PM, Kanter KR, McConnell M et al Adult congenital heart surgery: adult or pediatric facility? Adult or pediatric surgeon? Ann Thorac Surg 2009;87:833–40. 19231400 10.1016/j.athoracsur.2008.12.027

[15] Grover A , Gorman K, Dall TM, Jonas R, Lytle B, Shemin R et al Shortage of cardiothoracic surgeons is likely by 2020. Circulation 2009;120:488–94. 19635974 10.1161/CIRCULATIONAHA.108.776278

